# Continuous glucose monitoring metrics following sub-Tenon’s injection of triamcinolone acetonide for diabetic macular edema

**DOI:** 10.1007/s00417-023-06275-y

**Published:** 2023-10-21

**Authors:** Rei Sotani-Ogawa, Sentaro Kusuhara, Yushi Hirota, Kyung Woo Kim, Wataru Matsumiya, Wataru Ogawa, Makoto Nakamura

**Affiliations:** 1 Division of Ophthalmology, Department of Surgery, Kobe University Graduate School of Medicine, 7-5-1 Kusunoki-Cho, Chuo-Ku, Kobe, 650-0017 Japan; 2 Division of Diabetes and Endocrinology, Department of Internal Medicine, Kobe University Graduate School of Medicine, Kobe, Japan

**Keywords:** Continuous glucose monitoring, Corticosteroid, Diabetes, Diabetic macular edema, Hyperglycemia, Triamcinolone acetonide

## Abstract

**Purpose:**

This pilot study aims to comprehensively evaluate the effects of sub-Tenon’s injection of triamcinolone acetonide (STTA) on glycemic control in patients with diabetic macular edema (DME) using professional continuous glucose monitoring (CGM).

**Methods:**

This retrospective study analyzed changes in glycemic control in 20 patients with type 2 mellitus and DME following single STTA (20 mg/0.5 mL) using The FreeStyle Libre Pro system. Professional CGM provides core CGM metrics such as the percentage of time that glucose levels fall within a target range and include the time in range (TIR) (70–180 mg/dL), time above range (TAR) (> 180 mg/dL), and time below range (TBR) (< 70 mg/dL). Outcome measures were the changes in CGM metrics (TIR, TAR and TBR) and the percentage of patients in whom TAR increased by at least 10 percentage points (ppt) 4 days before to 4 days after STTA administration.

**Results:**

The mean CGM metrics (TIR/TAR/TBR) were 75.5%/19.9%/4.4% 4 days before STTA and 73.7%/22.4%/3.5% 4 days after STTA; the metrics 4 days before and 4 days after STTA were not significantly different (*P* = 0.625 for TIR, *P* = 0.250 for TAR, and *P* = 0.375 for TBR). TAR increased by more than 10 ppt in four (20%) patients treated with sulfonylurea and/or insulin.

**Conclusion:**

Although there were no significant changes in the CGM metrics, four patients developed CGM-measured hyperglycemia after STTA for DME.

**Supplementary Information:**

The online version contains supplementary material available at 10.1007/s00417-023-06275-y.


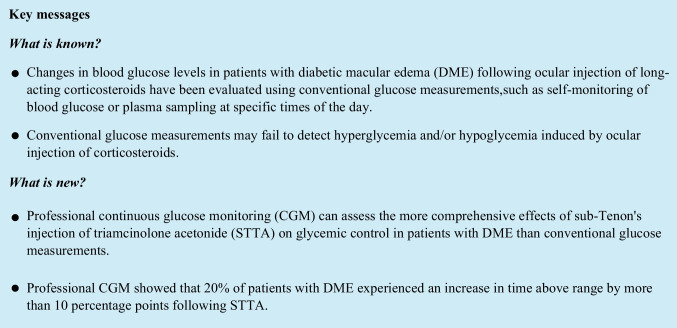


## Introduction/Background

The current standard treatment for diabetic macular edema (DME) is intravitreal injection of anti-vascular endothelial growth factor (VEGF) [1]. The DRCR.net Protocol I showed that in a subgroup analysis of pseudophakic eyes, the mean change in the best-corrected visual acuity (BCVA) in eyes treated with intravitreal triamcinolone acetonide (TA) injection + prompt laser therapy was comparable to those treated with intravitreal injections of an anti-VEGF drug (ranibizumab); however, this is not level I evidence[2]. Therefore, local injection of long-acting corticosteroids, such as TA, or dexamethasone implants is frequently and widely used as an alternative therapy for eyes with DME that are refractory to anti-VEGF therapy. However, local corticosteroid injection can cause systemic and ocular side effects. Data from previous studies suggest that ophthalmologists tend to predominantly monitor only for ocular side effects, including cataract formation, ocular hypertension, and bacterial endophthalmitis [3–5]. In previous studies focusing on glucose levels in patients with diabetes following ocular injection of long-acting corticosteroids for DME, changes in the blood glucose (BG) level were not comprehensively assessed because the investigators used self-monitoring of BG (SMBG) or plasma sampling at specific times of the day [6–9]. Accordingly, systemic side effects of local corticosteroid injection, such as abnormal BG elevation, are likely underreported or underestimated. Continuous glucose monitoring (CGM) provides a more comprehensive picture of glycemia than either SMBG or HbA1c [10, 11]. The FreeStyle Libre Pro system (Abbott Diabetes Care, Witney, United Kingdom) consists of a factory-calibrated sensor that continuously measures glucose levels in the interstitial fluid for up to 14 days (the maximum data acquisition time for a single sensor) and a reader that retrieves stored data required for an ambulatory glucose profile (AGP) report, which is a standardized statistical and graphical information that yields several CGM metrics and identifies patterns and trends in BG levels [12]. In the current pilot study, to assess the effects of sub-Tenon’s injection of TA (STTA) on glycemic control in a comprehensive manner, we utilized the pooled CGM data obtained in clinical practice and investigated the changes in CGM metrics in patients with DME following a single STTA.

## Method

### Study design

This retrospective study was approved by the institutional review board of the Kobe University Graduate School of Medicine (permission number: B210299) and adhered to the tenets of the Declaration of Helsinki (7^th^ revision). We performed a sequential review of the medical records of patients who met the inclusion criteria (glucose levels were assessed using the FreeStyle Libre Pro system 1 week before and after a single STTA for DME at Kobe University Hospital between January 2018 and November 2021). Data extracted from the medical records included the following: age, sex, eye laterality, axial length, lens status, stage of diabetic retinopathy, BCVA, intraocular pressure (IOP), central retinal thickness (CRT), macular volume (MV), history of vitrectomy, type of diabetes, HbA1c, plasma glucose (PG), C-peptide (CPR), C-peptide index (CPI), hypoglycemic drugs, systemic comorbidities, and CGM metrics. The exclusion criteria were patients with incomplete CGM data or those who had undergone prior CGM analysis.

### STTA

STTA was administered on an outpatient basis. After anesthetizing the ocular surface with 4% lidocaine eye drops, disinfection of the skin around the eye and ocular surface was performed using 5% povidone–iodine and eightfold diluted PA・IODO Ophthalmic and Eye washing Solution Disinfection (Nitten Pharmaceutical Co., Nagoya, Japan). An eye lid speculum was then placed, and STTA (20 mg/0.5 mL) (Kenacort–A, Bristol-Myers Squibb, Tokyo, Japan) was administered. After injection, antibiotic eye drops and ointment were administered, the lid speculum was carefully removed, and an eyepatch was placed. The patient was instructed to remove the eyepatch the next day and continue antibiotic instillation for 4 days.

### Professional CGM

A FreeStyle Libre Pro sensor was applied to the upper arm on one side one week prior to STTA administration. One week after STTA administration, the sensor, which holds data indefinitely, was removed, and data were downloaded and analyzed using the FreeStyle LibreView software. Consequently, we obtained data on core CGM metrics and AGP in each patient. The CGM metrics used in this study was the percentage of time that sensor glucose levels fell within a target range, including the time in range (TIR) (70–180 mg/dl), time above range (TAR) (> 180 mg/dL), and time below range (TBR) (< 70 mg/dL) as is evident from the consensus among diabetes specialists [12]. The FreeStyle Libre Pro system is a retrospective CGM system that allows patients and physicians to review the data only after wearing the sensor.

### Outcome measures

Primary outcome measures were the changes in CGM metrics (TIR, TAR, and TBR) and the percentage of patients in whom the TAR increased by at least 10 percentage points (ppt) 4 days before to 4 days after STTA. In order to account for the potential stress associated with sensor placement or STTA procedure, we have excluded data from at least the first two days following sensor attachment and from the day of STTA. Secondary outcome measures were the changes in the logarithm of the minimum angle of resolution (logMAR) BCVA, IOP, CRT, and MV from before STTA to 1 month after STTA. BCVA was measured using a standard Landolt chart, and the decimal BCVA values were converted to logMAR BCVA values for statistical analyses. CRT and MV values were automatically calculated by the built-in software using the Macular Cube 200 × 200 scan data acquired by optical coherence tomography (Cirrus HD-OCT 5000, Carl Zeiss Meditec, Tokyo, Japan).

### Statistical analyses

Values were described as medians with interquartile ranges (IQRs) unless otherwise indicated. The Wilcoxon test was used to compare the time of each variable. To compare the differences in variables between two groups (i.e., patients with and without increased TAR after STTA), the Mann–Whitney U test or Chi–square test was used as appropriate. Statistical analyses were performed using MedCalc v.20.027 software (MedCalc Software, Ostend, Belgium). *P*-values < 0.05 were considered significant.

## Results

Of the 23 patients who met the inclusion criteria, 3 were excluded due to incomplete CGM data. Accordingly, data from the remaining 20 patients were used for the analyses. The characteristics of the 20 patients are summarized in Table 1. The prescribed hypoglycemic drugs of the patients included dipeptidyl peptidase-4 inhibitors (85%), biguanides (65%), sulfonylurea (35%), insulin (15%), thiazolidinediones (10%), alpha-glucosidase inhibitors (10%), sodium-glucose cotransporter 2 inhibitors (10%), meglitinides (5%), and glucagon-like peptide-1 receptor agonist (5%). Systemic comorbidities included hypertension (45%), dyslipidemia (20%), chronic kidney disease (10%), angina pectoris (10%), and others (50%).

**Table 1:** Characteristics of patients with DME prior to STTA

Number of patients	20
Age (years), median (IQR)	72 (65.25, 76.25)
Sex (female), n (%)	9 (45)
Eye (right), n (%)	11 (55)
Axial length (mm), median (IQR)	23.44 (23.04, 24.24)
Lens status, n (%)	
Phakic	11 (55)
Intraocular lens	9 (45)
Retinopathy, n (%)	
Mild NPDR	1 (5)
Moderate NPDR	4 (20)
Severe NPDR	7 (35)
PDR	8 (40)
History of vitrectomy, n (%)	8 (40)
LogMAR BCVA, median (IQR)	0.222 (0.097, 0.429)
Intraocular pressure, median (IQR)	15 (14.75, 16.25)
Central retinal thickness (µm), median (IQR)	495.5 (414.75, 570.25)
Macular volume (mm^3^), median (IQR)	12.35 (11.525, 13.8)
Plasma glucose (mg/dL), median (IQR)	133.5 (111, 161)
HbA1c (%), median (IQR)	7.1 (6.55, 7.6)
CPR (ng/mL), median (IQR)	2.37 (1.63, 4.12)
CPI, median (IQR)	1.79 (1.19, 2.78)

*Abbreviations*: *DME* diabetic macular edema, *STTA* sub-Tenon’s injection of triamcinolone acetonide, *IQR* interquartile range, *NPDR* non-proliferative diabetic retinopathy, *PDR* proliferative diabetic retinopathy, *logMAR* logarithm of minimum angle of resolution, *BCVA* best-corrected visual acuity, *CPR* C-peptide, *CPI* C-peptide index

**Fig. 1:**
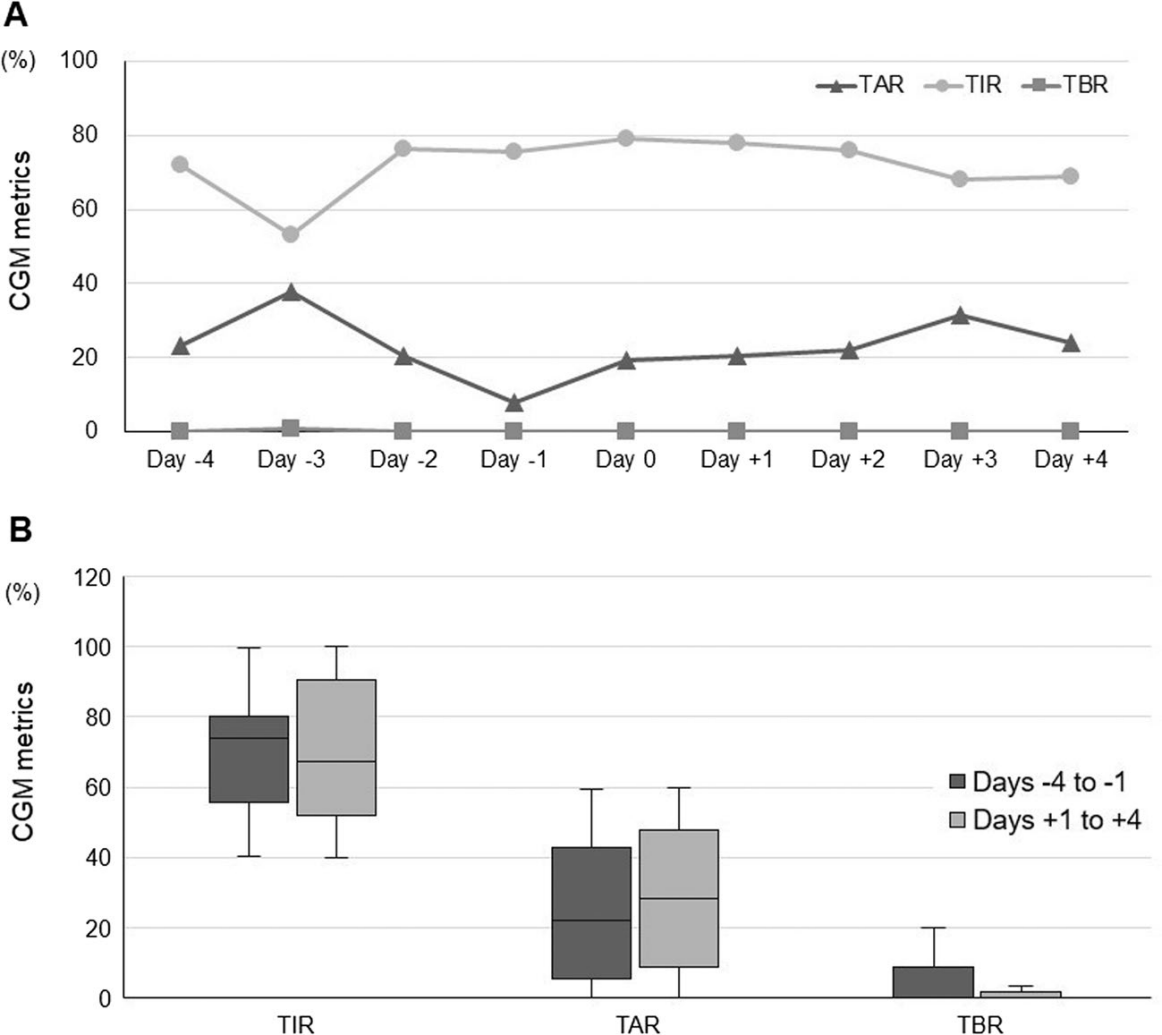
Changes in CGM metrics following STTA for DME. **A** A line graph shows changes in the median CGM metrics over time following STTA administration for DME. **B** A box plot comparing CGM metrics between days − 4 to − 1 and days + 1 to + 4. A significant change was not observed in each CGM metric (*P* = 0.625 for TIR, *P* = 0.250 for TAR and *P* = 0.375 for TBR). *CGM*, continuous glucose monitoring; *STTA*, sub-Tenon’s injection of triamcinolone acetonide; *DME*, diabetic macular edema; *TIR*, time in range; *TAR*, time above range; *TBR*, time below range

Changes in CGM metrics over time are shown in Fig. 1. The median (IQR) TIR (%) was 72% (56.75%, 82%) on day − 4, 53% (48.75%, 86%) on day − 3, 76.5% (59.5%, 82%) on day − 2, 75.5% (70%, 92%) on day − 1, 79% (65%, 87.5%) on day 0, 78% (45%, 93%) on day + 1, 76% (52%, 93%) on day + 2, 68% (50%, 89.75%) on day + 3, and 69% (52%, 83.25%) on day + 4 (the date of STTA administration was counted as day 0). The median (IQR) TAR (%) was 23.5% (3.25%, 43.25%) on day − 4, 38% (11%, 50.25%) on day − 3, 20.5% (10.25%, 33.5%) on day − 2, 8% (2%, 29.25%) on day − 1, 19.5% (9.25%, 35%) on day 0, 20.5% (5.75%, 55%) on day + 1, 22% (7%, 48%) on day + 2, 31.5% (8.75%, 41.5%) on day + 3, and 24% (10.5%, 36.5%) on day + 4. The median (IQR) TBR (%) was 0% (0%, 0.5%) on day − 4, 1% (0%, 4%) on day − 3, 0% (0%, 7.25%) on day − 2, 0% (0%, 4.75%) on day − 1, 0% (0%, 0%) on day 0%, 0% (0%, 3%) on day + 1, 0% (0%, 0.25%) on day + 2, 0% (0%, 0.75%) on day + 3, and 0% (0%, 0%) on day + 4. There was not a significant difference between days − 4 and − 1 and between days + 1 and + 4 in each CGM metric (*P* = 0.625 for TIR, *P* = 0.250 for TAR, and *P* = 0.375 for TBR) (see Supplementary Materials).

**Fig. 2:**
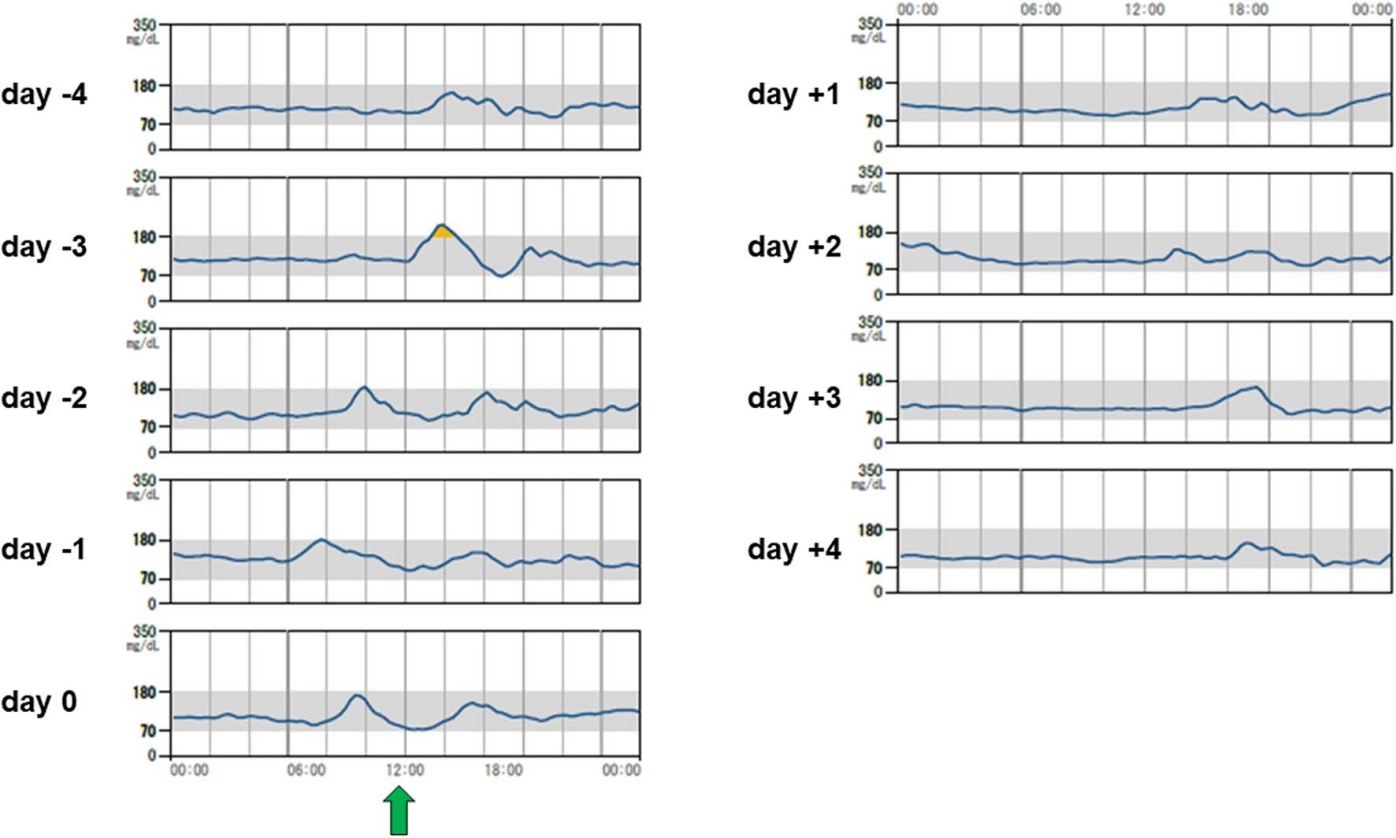
Daily glucose profiles of a representative case without increased TAR following STTA. Blood glucose levels remained within normal range even after STTA administration. A single green arrow indicates the time point when STTA was performed. *TAR*, time above range; *STTA*, sub-Tenon’s injection of triamcinolone acetonide

**Fig. 3:**
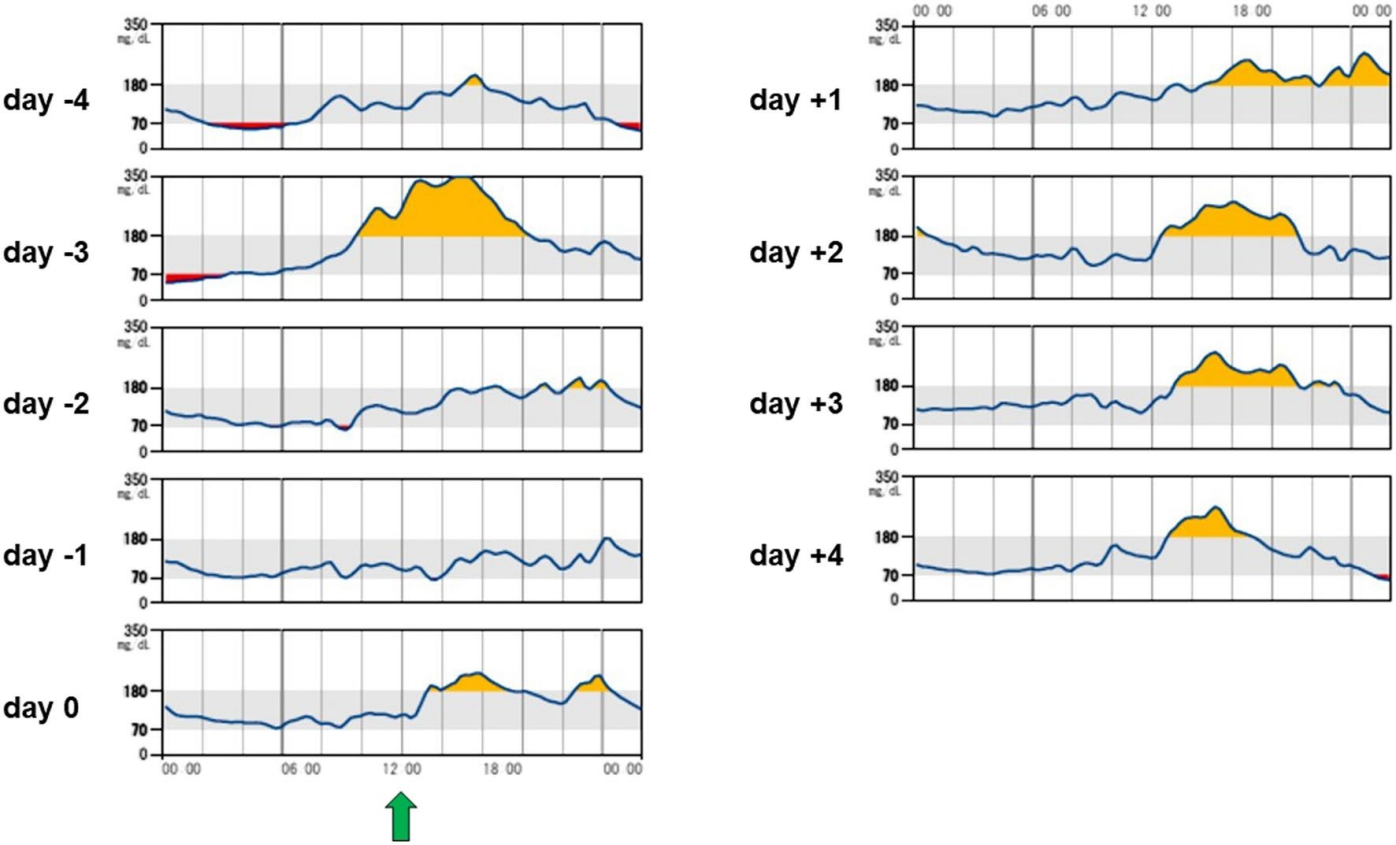
Daily glucose profiles of a representative case with increased TAR following STTA. Blood glucose levels were elevated above normal range predominantly in the afternoon and evening after STTA. A single green arrow indicates the time point when STTA was performed. *TAR*, time above range; *STTA*, sub-Tenon’s injection of triamcinolone acetonide

The TAR in four (20%) patients increased by more than 10 ppt from four days before to four days after STTA; all were maintained on sulfonylureas and/or insulin. The characteristics of these patients are shown in Table 2. In the comparison of variables between the increased TAR group (*n* = 4) and non-increased TAR groups (*n* = 16), a significant difference was observed in age, sex, and insulin use. The median (IQR) age was 77 (76.75, 77.75) and 71 (63, 73.25) years in the increased and non-increased TAR groups, respectively (*P* = 0.014). Women comprised 75% and 50% of the patients in the increased and non-increased TAR groups, respectively (*P* = 0.038). The proportion of patients who used insulin in the increased and non-increased TAR groups was 50% and 6%, (*P* = 0.033). The median (IQR) of other variables in the increased and non-increased TAR groups was 7.85% and 7.00% for HbA1c (*P* = 0.105), 169 mg/dL and 126 mg/dL for PG (*P* = 0.219), 1.85 ng/mL and 2.37 ng/mL for CPR (*P* = 0.643), and 0.896 and 1.877 for CPI (*P* = 0.254), respectively. The proportion of patients who used sulfonylureas in the increased and non-increased TAR groups was 50% and 31%, respectively (*P* = 0.493). The daily glucose profiles of representative cases with or without increased TAR following STTA are presented in Figs. 2 and 3. Hyperglycemia occurred in the afternoon and evening in three (75%) patients with increased TAR.

**Table 2:** Characteristics of patients with increased TAR following STTA

No	Increment of TAR (ppt)	Age range (years)	Sex	HbA1c (%)	CPR (ng/mL)	CPI	SU dose (mg/day)	Insulin dose (U/day)	Comment
1	16.5	80 s	Male	8.4	0.41	0.27	0	34	
2	17.0	70 s	Male	8.7	2.96	1.08	4	0	
3	20.0	70 s	Female	7.3	10.35	5.51	6	0	During hormone therapy for neuroendocrine tumor
4	12.5	70 s	Male	6.8	0.74	0.71	0	23	

*Abbreviations*: *TAR* time above range, *STTA* sub-Tenon’s injection of triamcinolone acetonide, *ppt* percentage point, *CPR* C-peptide, *CPI* C-peptide index, *SU* sulfonylurea

One month after STTA administration, the median (IQR) logMAR BCVA, IOP (mmHg), CRT (µm) and MV (mm^3^) was 0.261 (0.128, 0.429), 16 (15.1, 16.8), 385 (307.5, 486.25), and 12.2 (11.275, 13.5), respectively. There was a significant difference in the CRT before and 1 month after STTA (*P* < 0.001), but not in the logMAR BCVA (*P* = 0.326), IOP (*P* = 0.190), and MV (*P* = 0.105).

## Discussion

In the current study, CGM metrics (TIR, TAR, and TBR) did not significantly change after STTA, indicating that the overall effect of STTA on glycemic control is modest. The unchanged glycemic control after STTA in this study is consistent with those previously reported [6]. Feldman-Billard et al. analyzed BG changes in 67 patients with DME after a single 4-mg intravitreal TA injection by monitoring 6–10 daily capillary BG measurements and concluded that a 4-mg intravitreal TA injection would not generally change glycemic levels in patients with diabetes [6]. Kadeli et al. prospectively evaluated the influence of STTA (40 mg) on PG (fasting PG [FPG] and HbA1c), cortisol, and adrenocorticotrophic hormone (ACTH) in 33 patients with type 2 diabetic mellitus and DME; they found that STTA did not significantly change the FPG, HbA1c, cortisol, and ACTH levels as compared with those in the controls (ST injection of saline solution) [8]. Additionally, Posch-Pertl et al. assessed the PG profiles of nine patients with type 2 diabetes mellitus and DME who had been implanted with intravitreal dexamethasone (0.7 mg) and reported that glucose levels using a 7-point PG profile did not show a significant change after intravitreal dexamethasone implantation at any single time point (1–3 days, 1 week and 1 month) [9]. However, in a clinical trial evaluating glucose levels following intra-articular injection of TA crystalline suspension in patients with type 2 diabetes mellitus with knee osteoarthritis using CGM, the mean daily CGM-measured glucose level increased from baseline (days − 3 to − 1) to days 1–3 by 37.1 mg/dL (from 161.7 mg/dL to 198.8 mg/dL), suggesting that CGM could assess changes in glucose levels induced by local TA injection that conventional SMBG may fail to reveal [13].

Notably, 20% of the patients had a greater than 10 ppt increase in TAR following STTA in the current study. As TAR is a metric that includes time, it could more accurately reflect BG changes than repeated SMBG. As mentioned above, patients with type 2 diabetes and knee osteoarthritis exhibited increased daily CGM-measured glucose levels following intra-articular TA injection [13]. Additionally, the daily glucose profiles of patients with increased TAR in our study showed hyperglycemia in the afternoon and evening. This pattern of hyperglycemia is consistent with that reported by Burt et al., who investigated the circadian effects of prednisolone on glucose concentrations in hospitalized patients with chronic obstructive pulmonary disease without known diabetes using CGM [14]. The elevation of glucose levels after STTA was previously reported. Toda et al. analyzed FPG levels before vs. after STTA in 16 patients with type 2 diabetes and DME and found that 3 (19%) patients had FPG levels equal to or greater than 200 mg/dL after administration [7]. Given the clinical impact of hyperglycemia, it should be noted that the overall effect of STTA on glycemic control is not significant; however, some patients may develop hyperglycemia after STTA. The daily glucose profiles of the representative case with increased TAR following STTA (Fig. 3) clearly shows that SMBG values at specific time points would over- or under-estimate the impact of STTA on the BG level. If a blood sample was taken at a time when the glucose level is close to its peak, an internist who was not informed of previous STTA from an ophthalmologist might decide to shift to a more aggressive diabetes management strategy, which may lead to severe hypoglycemia as the effects of STTA subside with time. Although we searched for predictors of cases with increased TAR following STTA, the number of cases was insufficient to perform a logistic analysis. However, the insulin secretion capacity might be relevant to this event because all four patients with increased TAR were treated with sulfonylureas and/or insulin, and three (75%) had a CPI < 1.1. Meanwhile, one patient was undergoing hormone therapy for a neuroendocrine tumor, and the excess steroid hormones may have led to the increased TAR.

We had no alternative but to conduct a small retrospective study this time because no preceding studies have not been conducted so far. Therefore, most of the limitations in this study stem from its retrospective nature and the small sample size. First, the sample size was probably too small to assess the statistical significance of the rare side effects (i.e., CGM-measured hyperglycemia after STTA) although we do not thick that this detracts from the clinical relevance of this study. Second, it is unknown whether intravitreal TA injection or an intravitreal dexamethasone implant for DME shows similar daily CGM-measured glucose levels as those with STTA. Third, we did not determine the reproducibility of the daily glucose profiles following STTA. Fourth, the threshold level in TAR increase does not have a basis in the evidence and should, therefore, be subject to validation in future studies. Finally, the maximum duration of sensor use of the current Libre Pro device is 14 days; therefore, the long-term effects of STTA on daily glucose profiles remains unknown. Further prospective analyses and advances in CGM technology could resolve these problems and contribute to highly accurate predictions of blood glucose fluctuations.

In conclusion, we analyzed CGM data from 20 patients with DME following a single STTA and obtained novel insights into the changes in glucose levels that conventional SMBG failed to reveal. STTA for DME does not significantly change the CGM metrics. However, it should be noted some patients may develop CGM-measured hyperglycemia after STTA.

### Supplementary Information

Below is the link to the electronic supplementary material.

Supplementary file1 (DOCX 19 KB)
