# Supplementary material for: Continuous glucose monitoring metrics following sub-Tenon’s injection of triamcinolone acetonide for diabetic macular edema
Source: Graefes Arch Clin Exp Ophthalmol. 2023 Oct 21;262(2):449–56. doi: 10.1007/s00417-023-06275-y (PMC10844153; doi:10.1007/s00417-023-06275-y)
Supplement: Supplementary file 1 — Supplementary file1 (DOCX 19 KB) [file 417_2023_6275_MOESM1_ESM.docx]

**Supplementary Table 1.** Changes in CGM metrics over time following STTA

|  | Day -4 | Day -3 | Day -2 | Day -1 | Day 0 | Day +1 | Day +2 | Day +3 | Day +4 |
| --- | --- | --- | --- | --- | --- | --- | --- | --- | --- |
| TAR (%) | 23.5  (3.25, 43.25) | 38  (11, 505) | 20.5  (10.25, 33.5) | 8  (2, 29.25) | 19.5  (9.25, 35) | 20.5  (5.75, 55) | 22  (7, 48) | 31.5  (8.75, 41.5) | 24  (10.5, 36.5) |
| TIR (%) | 72  (56.75, 82) | 53  (48.75, 86) | 76.5  (59.5, 82 | 75.5  (70, 92) | 79  (65, 87.5) | 78  (45, 93) | 76  (52, 93) | 68  (50, 89.75) | 69  (52, 83.25) |
| TBR (%) | 0  (0, 0.5) | 1  (0, 4) | 0  (0, 7.25) | 0  (0, 4.75) | 0  (0, 0) | 0  (0, 3) | 0  (0, 0.25) | 0  (0, 0.75) | 0  (0, 0) |

Data are provided as median (inter-quartile ranges)

Abbreviations: CGM, continuous glucose monitoring; STTA, sub-Tenon’s injection of triamcinolone acetonide; TAR, time above range; TIR, time in range; TBR, time below range
